# Hints Respecting the Hydrocephalus Internus

**Published:** 1788

**Authors:** John Warren

**Affiliations:** Physician at Taunton, honorary Member of the Medical Society at Edinburgh, Corresponding Member of the Royal Medical Society at Paris, and an Associate of the Academies at Bologna, Florence, and Rome


### III. *Hints respecting the Hydrocephalus internus; communicated in a Letter to Dr.* Simmons *by* John Warren, *M. D. Physician at Taunton, honorary Member of the Medical Society at Edinburgh, corresponding Member of the Royal Medical Society at Paris, and an Associate of the Academies at Bologna, Florence, and Rome.*

IT has frequently occurred to me, as a melancholy reflection, that many of the new and active remedies introduced into the practice of physic, have, after a time, by the improper and indiscriminate use of them, not only lost the reputation they had acquired in the hands of their inventors, but have been productive of so much mischief that it might possibly have been as well for society at large, had such remedies never been discovered.

I have been particularly induced to make this observation from having frequently witnessed the injurious effects of mercury, when administered as a remedy for a supposed hydrocephalus internus, which has existed only in the imagination of the practitioner.

The discovery of any means for the cure of so fatal a disease certainly merits the highest applause; and if the case described by the late

Dr. Dobſon, in the ſixth volume of the London Medical Obſervations and Inquiries, was truly a caſe of this kind, he is entitled to the warmeſt thanks of the public for the ſagacity he has diſplayed in inveſtigating the powerful effects of mercury, as a remedy for a complaint, which, till that period, ſeems to have been almoſt uniformly fatal.

The late very worthy and reſpectable Dr. Whytt candidly confeſſed, that of twenty caſes which had fallen under his obſervation, and in which the characteriſtic ſymptoms of this diſorder were preſent, he had not been fortunate enough to cure a ſingle patient. Dr. Percival, Dr. Haygarth, and ſome other very reſpectable phyſicians have, however, borne teſtimony to the ſucceſs of Dr. Dobſon's mode of treatment, and the teſtimony of ſuch men is not to be doubted, but with the greateſt diffidence. It would afford me infinite ſatisfaction to be able to give my ſuffrage alſo to the ſalutary effects of this plan of cure, but I muſt candidly acknowledge that I have no reaſon to ſpeak favorably of it from my own experience. In ten caſes of this deplorable malady which have fallen under my care, and in which the mercurial treatment was purſued, the event proved fatal. This want of fuccefs might, poffibly, be attributed to the mercury having, in each inftance, failed of producing the effect of falivation, which by fome may probably be deemed effential to its beneficial operation. That it fhould uniformly have failed of producing ptyalifm, has been matter of fome furprife to me; particularly as the mercurial courfe was ufed in the moft liberal manner with all my patients; in many cafes for feveral days together, to the amount of three or four grains of calomel every eight hours, without any purgative effect; befides a friction of a drachm of unguentum mercuriale fortius morning and evening; and this in children of fix, eight, or ten, years of age. Bliftering, it is true, was at the fame time employed with the greateft freedom in all the cafes; and bleeding was likewife ufed in feveral of them, at the commencement of the difeafe, for reafons which will hereafter be affigned; but I do not conceive, thefe means could have operated in counteracting any falutary effects which might otherwife have been expected from the mercurial courfe. In many of the cafes above alluded to, I had, after death, an opportunity of examining the ftate of the brain, when the caufe of the fatal termination was afcertained, beyond a doubt, to be water lodged lodged, in greater or ſmaller quantity, in the ventricles of that organ.

This general failure of ſucceſs on my part, has almoſt ſtaggered my faith in the remedy itſelf. At any rate it has led me to ſuppoſe, that the diſeaſe has not really exiſted in all thoſe caſes, which are ſaid to have been cured by the uſe of mercury. I am the more ſtrengthened in this concluſion from obſerving in the account of many of thoſe caſes, that a ſalivation was very eaſily excited; whereas from what has been above advanced, it would appear that this effect of mercury is, at any rate, with the greateſt difficulty obtained, even when that remedy is employed in enormous quantities. Nor can this be altogether wondered at, when we reflect on the general torpor and inertia that prevail univerſally in the ſyſtem in theſe caſes; and which evidently appear from the ſlowneſs of the pulſe, the remarkably tardy ſtate of the bowels, and the long retention of urine to which ſuch patients are liable.

But again, if mercury be ſo ſingularly beneficial in this diſeaſe, as many of its abettors would induce us to ſuppoſe, it would appear that I have been peculiarly unfortunate in my practice; as in the account of many of the caſes publiſhed, publiſhed, we are told that it is not always neceſſary to the cure, that the mercurial treatment ſhould be puſhed to the length of ſalivation, for it has been frequently found ſufficient only to ſaturate the ſyſtem with it.

If this be true, that ſimply ſaturating the ſyſtem with mercury is ſufficient to effect a cure, Dr. Dobſon's theory of its action muſt be without foundation. For he ſays he was firſt induced to make a trial of it, from an idea "that mercurials ſo far urged as to enter into the courſe of the circulation, and affect the ſalivary glands, might poſſibly reach the ſyſtem of abſorbents in the ventricles of the brain, and thus remove the extravaſated fluid."

Although I can by no means imagine it likely that the gentlemen already mentioned, as well as ſome other eminent phyſicians, who have expreſſly written on this ſubject, would readily impoſe on themſelves, in believing they had effected cures of a diſeaſe which really never exiſted; yet I am by no means ſingular in my ſuppoſition, that practitioners at large have frequently erred in this reſpect; and I know ſome, who have acquired no inconſiderable degree of credit by offering to the public ſuch ſpurious accounts of cures performed by them. It is, however, however, by no means my intention to inſinuate here, that ſuch perſons have deſignedly been guilty of an impoſition; their only error has been in deceiving themſelves, by imagining they had been more ſucceſsful than their neighbours; when they have cured ſome other diſtemper which they had miſtaken for the hydrocephalus internus. Indeed it is not much to be wondered at, that practitioners who are but little converſant with the diſeaſe, ſhould ſo eaſily err in this reſpect, as ſo many of the ſymptoms that accompany a dropſy of the brain are frequently attendant on worm caſes, dentition, and other irritating cauſes, that it is difficult to fix on any which can particularly chataсteriſe it.

In two caſes that have fallen under my obſervation every ſymptom of the hydrocephalus internus was prevalent; but on diſſection the following phenomena preſented themſelves. On opening the head of one of the patients, an effuſion of red blood to the quantity of nearly two ounces was found lodged on the pia mater immediately under the temporal bone; and in the other, a ſteatomatous tumor of the ſize of a cheſnut was diſcovered in the body of the brain. No collection of water appeared in either caſe. In both theſe children even the ſymptom of

ſtrabiſmus

ſtrabiſmus * or ſquinting, which has been heretofore held pathognomonic of the hydrocephalus internus, took place.

But ſuch miſtakes as the following are altogether inexcuſable, and it is from an apprehenſion that numberleſs ſimilar ones have already happened, and may in future occur, that I have principally been induced to turn my thoughts to the ſubject of the preſent paper.

It is not many years ſince I was requeſted to viſit a gentleman who had been reduced to a ſtate of extreme danger in conſequence of a ſalivation excited and kept up for

* I am thoroughly perſuaded that the ſtrabiſmus which the ancients conſidered as a principal pathognomonic ſymptom of this diſeaſe by no means merits that appellation, as it has never occurred to me earlier than two or three days before the death of the patient, and in other grievous affections of the brain, the ſame ſymptom has been obſerved to take place before that time. It has been alledged indeed by many reſpectable phyſicians that the ancients were in a great meaſure ignorant of this diſeaſe, at leaſt of that ſtate of it depending on a ſurcharge of ſerous fluids in the ventricles of the brain; and this idea, I ſuppoſe, muſt have ariſen from Celſus taking no notice of it, and from Galen mentioning it in ſuch a ſtyle, as if he knew nothing concerning it; but that Hippocrates was perfectly acquainted with it, appears, I think, clearly from the following for ſeven weeks together, to carry off, as his apothecary termed it, a ſerous effuſion on his brain; when, in fact, his diſeaſe was no other than a hemicranium, which was afterwards cured by the Peruvian bark and Valerian.

A young lady alſo of my acquaintance was through a ſimilar miſtake reduced to the laſt extremity, by a long and ſevere courſe of mercury adminiſtered, under an idea, that her caſe was hydrocephalous; when in fact it was of the kind we denominate *nervous*, attended with a lowing enumeration of ſymptoms, in his treatiſe on diſeaſes:

Ἢν ὕδωρ ἐπὶ τῷ ἐγκεφάλῳ γένηται, ὀδύνη ὀξείη ἴσχει διά τοῦ βρέγματος καὶ τῶν κροτάφων ἔνιοτε ἄλλη. καὶ ῥίγος καὶ πυρετός ἄλλοτε καὶ ἄλλοτε. καὶ τὰς χώρας τῶν ὀφθαλμῶν ἀλγέει καὶ ἀμβλυώσσει. καὶ ἡ κόρη σχίζεται καὶ δοκέει ἐκ τοῦ ἑνὸς δύο ὁρᾷν. καὶ ἢν ἀναστῇ, σκοτοδινίη μὶν λαμβάνει. καὶ τὸν ἄνεμον οὐχ ἀνέχεται, οὐδὲ τὸν ἥλιον καὶ τὰ ὦτα τέτριγε. καὶ τῷ ψόφῳ ἄχθεται ἀκȣ́ων. καὶ ἐμέει σίελα καὶ λάπην, ἐνίοτε δὲ καὶ τά σιτία. *i. e.* Aqua, ſi in cerebro ſuborta fuerit, dolor acutus ſinciput & tempora, interdumque alias capitis partes, detinet, ſubindeque rigor & febris; oculorum regiones dolor occupat; iique caligant, pupilla ſcinditur, & ex uno, duo ſibi cernere homines videntur, & ſi quis ſurrexerit, ipſum tenebræ prehendunt, neque ventum, neque ſolem ſuſtinet, aures tinniunt, ſalivam & pituitam vomitione refundit, quandoque vero etiam cibos.— HIPPOC. de Morb. Lib. II. Sect. V.

 violent violent periodic head-ach, which was afterwards remedied by an antiſpaſmodic and tonic courſe.

But the tragic termination of the following caſe is ſuch as ſeriouſly to intereſt the feelings of humanity.

A young gentleman, about three years and a half old, and of a delicate conſtitution, was attacked in the month of September with pains in his ſtomach and bowels, and ſlight ſymptoms of fever; but as he was capable of amuſing himſelf, and of being amuſed, little attention was paid to his complaints for the firſt ſix or ſeven days. About this time the pains in his bowels became more urgent, and he had frequently ſlimy and green evacuations by ſtool. Severe efforts to vomit now likewiſe often took place; his fever increaſed conſiderably; he complained much of head-ach, was drowſy, and withal extremely reſtleſs. On the evening of the ninth day of the diſeaſe, when the little patient was labouring under the above ſymptoms, the family apothecary (ſince dead) was requeſted to attend. As the child was reſtleſs, nothing was thought to be more likely to afford relief than a draught containing ſix drops of liquid laudanum and ten grains of cordial confection. This medicine was accordingly adminiſtered, but the next next morning every ſymptom was exaſperated. The child had had no ſtool during the night, and the vomiting had ceaſed (both of which evacuations had before conſtantly given relief), and the patient from being drowſy was become comatoſe. The diſeaſe was now pronounced to be a dropſy of the brain, and the anxious parents were informed that a mercurial courſe was alone capable of affording a poſſibility of relief. Half a drachm of the ſtrong mercurial ointment was immediately rubbed on each leg; but, moſt unfortunately for the child, no calomel was given internally, fiom an apprehenſion of its irritating effects on the bowels. The friction was repeated in the evening to the ſame amount as before, and twice the day following. On the morning of the third day, a ſlight ptyaliſm made its appearance, but with no alleviation of the ſymptoms, which had been rapidly getting worſe during the whole courſe. It was hoped a palliation of them would take place on the ſalivation being increaſed; recourſe was accordingly had to another friction; the ptyaliſm now became profuſe, and on the day following, ſo great was the irritation produced, that the child was ſeized with univerſal ſpaſms, which continuing with unabated violence for ſeveral hours, at length cloſed the mournful ſcene.

The head was opened, and to the aſtoniſhment of the attendants, no water was diſcovered; but on examining the firſt paſſages, they were found replete with viſcid matter, and with crudities blended with an unuſual quantity of bile. They were likewiſe in many places in a ſtate of inflammation.

This child's complaint was ſimply a fever of the ſeaſon of the remittent kind, depending on a diſeaſed ſtate of the firſt paſſages; and if theſe had been properly evacuated, in all probability, a cure would have been eaſily and ſpeedily completed: nor is it at all unlikely, had a few doſes of calomel been adminiſtered, on a preſumption of the caſe being hydrocephalus, that the patient might have recovered, and the mercurial courſe have obtained the credit of curing a diſeaſe which is moſt commonly irremediable.

Do not ſuch egregious errors, as theſe, call aloud for animadverſion; and are they not proofs of the remark I advanced, at the commencement of this paper, of the injurious effects of active medicines, when in the hands of thoſe who do not poſſeſs ſufficient judgment to diſcriminate diſeaſes?

It is to be lamented that ſuch men, before they they venture to adminifter remedies for a fup-pofed hydrocephalus internus, do not make themfelves acquainted with the nature, fymp-toms and progrefs of the difeafe; an account of which, being accurately delineated, I fhall here tranfcribe, for their information, from the late Dr. Fothergill's paper on this fubject in the Medical Obfervations and Inquiries*.

"In moft of thofe whom I have feen in this "diftemper, a pain in fome part or other "below the head was the firft thing they com-"plained of; moft commonly about the nape "of the neck and fhoulders, often in the legs, "fometimes in the arms, but more rarely.

"This pain was not always alike acute, nor "always fixed to one place; fometimes it "feemed not to affect any of the limbs. In "thefe cafes, the head and ftomach feemed to "be more difordered; and indeed were always "difordered more or lefs from the beginning, "as far as I could learn. When the pain "was in the limbs, the ficknefs or head-ach "was lefs; when the head became the feat of "complaint, the pain in the limbs was feldom "or ever mentioned: fome had very violent "fickneffes, and violent head-achs alternately.

* Vol. IV. Page 45.

"From

" From being perfectly well and ſportive, " ſome were ſeized with theſe pains in the " limbs, or with ſickneſs, or head-ach ſlightly, " in a few hours, commonly, after dinner. " Some have been obſerved to droop a few days " before they complained of any part being " much indiſpoſed. In this manner they con- " tinued three, four, or five days, more or " leſs, as the children were healthy and vigo- " rous, when the diſtemper begins to ſhow it- " ſelf in an alarming manner.

" They then commonly complain of a moſt " acute pain in the head, deep ſeated, and ex- " tending acroſs the forehead from temple to " temple. They are generally very ſick be- " tween whiles, crying out in the moſt affect- " ing manner, *Oh, my head! Oh, I am ſick!* " alternately, and with ſhort intervals: " doſing a little in theſe intervals, breathing " irregularly, and ſighing much while awake. " Sometimes they only ſeem to breathe in ſighs " for ſome minutes together.

" The pulſe, from being regular as in health, " as the diſeaſe creeps on, becomes irregular; " ſlower, for the moſt part, at firſt than it " ought to be; it grows ſtill ſlower as the pain " increaſes, gradually likewiſe irregular, the

" ſtrokes

" strokes being made both with unequal force " and in unequal times. The limbs, for the " most part, are temperate, in respect to heat, " after the first access, which is often attended " with feverish heats, especially towards eve- " ning and forepart of the night, and till with- " in a day or two of their dissolution; the " pulse then becomes extremely quick, the " breathing deep, irregular, and laborious, " the heat exessive and more general. The " head is always hot from the first attack, and " the *præcordia* likewise.

" Almost every symptom that is known to " attend an irritating cause existing in the " brain, appears in its turn; first, pain in the " limbs, sickness, and head-ach.

" Short disturbed sleeps, startings, irregu- " lar pulse, watchfulness, and the pupils of " the eyes dilated.

" They are unwilling to be disturbed for any " purpose, are averse to light, take things " greedily, and cannot bear any posture but " that of lying horizontally. They attend less " to objects; when asleep, great part of the " whites of the eyes are seen, and are undis- " turbed by any thing but moving them. " Their urine comes away insensibly, and their

" stools

" ftools likewife. They often fcream out moft
" piercingly, but complain of nothing. One
" or both hands are moft commonly about
" their heads. At length the eyelids become
" paralytic, the *iris* immoveable: it gives them
" no apparent uneafinefs, if one raifes the
" eyelids with one's finger two or three days
" before they die. The heat of the head and
" trunk become exceffive; a great heat and
" fweat fpreads over the whole body, refpira-
" tion is altogether fufpirious, the pulfe trem-
" bling, and quick beyond the poffibility of
" counting, and the patient goes off gradually
" as the ftrength fails; fometimes a fpafm
" finifhes the cataftrophe."

To have rendered the enumeration of fymptoms complete, Dr. Fothergill fhould have taken notice of that ftate of the pulfe which frequently occurs at the beginning of the complaint, indicating an inflammatory diathefis, perhaps prefent in the head, and of courfe the ufe of phlebotomy. It is obferved by Dr. Fothergill in the above recital of fymptoms, that the pains in the limbs, inceffant head-ach and ficknefs, feem to be the moft certain intimations of danger. Thefe happen in other difeafes of children, but neither fo uniformly nor fo laftingly. Another circumftance, ftance, he fays, is likewife familiar, if not peculiar to this difeafe; for he never recollects one inftance in which the patient was not coftive, and in which, likewife, it was not without fingular difficulty that ftools were procured.

The difeafe alfo, as Dr. Whytt has obferved, may, in general, with propriety be divided into three ftages, principally denoted by the variations of the pulfe.

In the firft ftage, on the acceffion of the complaint, in children of tolerably firm conftitutions, the pulfe is regular and natural; but in the courfe of a few days it becomes full, ftrong and quick, fometimes rifing to 140 beats in a minute.

The fecond ftage commences with a change of the pulfe from a quick to a flow, irregular one; it at this time alfo lofes its ftrength and fulnefs, and its fall is from 120, 130 or 140 beats in a minute, to the natural ftandard, and, frequently, much below it.

In the third ftage, the pulfe again rifes confiderably, and fometimes very fuddenly; thus from 65 I have feen it rife to 90, 100, and from 100 to 130 or 140; and a little before death, I have known it amount to 200 beats, or upwards, in a minute.

As it was obferved above that the fymptoms of this difeafe are fo fimilar to thofe frequently excited by worms, it will, in all inftances, be prudent, on the firft fufpicion of its approach, immediately to have recourfe to fome mercurial purgative, which may probably be an effectual means of alleviating the fymptoms, fhould they originate from worms or other irritating caufes in the primæ viæ; and can do no injury fhould the primary affection be feated in the brain.

Although it was herétofore doubted, and ftill may be, whether a cure of the hydrocephalus internus can ever be expected, in confequence of no lymphatic veffels having been difcovered in the brain, yet I fee no reafon for fuch an opinion, independent of the cures of it which are faid lately to have been performed. It is true that no lymphatic veffels have, as yet, been certainly difcovered in this organ; but there can be little doubt that an abforption really takes place there, and if not performed by the common lymphatics, it is probable that the extremities of the red veins may anfwer the fame purpofe.

Independent then of any immediate communication between the ventricles of the brain and the ſalivary glands, agreeably to Dr. Dobſon's theory, may not the reputed beneficial effects of mercury, in caſes of hydrocephalus, be accounted for from its ſtimulating properties; ſince we know that medicines of this claſs are the moſt effectual of any for exciting abſorption. In this point of view would not the frequent uſe of gentle emetics (for emetics are known to be highly ſalutary in promoting abſorption from cavities), be a probable means of affording relief? They would certainly be much ſafer than mercury, their effects being only temporary.

It has been ſuppoſed, by Dr. Whytt, that this diſeaſe is frequently forming for many months, previouſly to its exciting any degree of attention, and that afterwards it may go on for many weeks before it comes to its fatal termination. Dr. Fothergill, on the contrary, informs us, that, when it has once taken place, it makes a rapid progreſs, and that he has ſeldom been able to trace it above three weeks from its commencement, to the tragic event.

My own experience inclines me to ſubſcribe to the former of theſe opinions; and I have obſerved, that in children of a puny and delicate conſtitution, the diſorder is generally much ſlower and leſs violent in its approach than in thoſe of firmer and more robuſt habits, although the unhappy victims of it have commonly been children of the rudeſt health, and of the moſt active and lively diſpoſitions.

This fact, ſimply ſtated as ſuch, would appear very much to countenance the ideas of the ingenious Drs. Withering and Quin as to the cauſe of the diſeaſe, which in their opinion originates from inflammation, and that the water found in the ventricles of the brain after death, is the conſequence and not the cauſe of the illneſs. That ſerous effuſions are often the conſequence of inflammation, will not admit of a doubt; and from my own obſervation on diſſecting hydrocephalous bodies, I believe them to be frequent cauſes of this diſorder: but why not ſuppoſe the diſeaſe, at times, likewiſe to be founded on an hydropic diatheſis, and a conſequent laxity of the exhalants; particularly in caſes where the conſtitution has been previouſly delicate, where the pain of the head has not been acute at the commencement, and where the pulſe has, from the firſt, indicated a ſtate oppoſite to that of inflammation?

Upon

Upon the whole, as I truſt I have ſhewn how extremely injurious the indiſcriminate uſe of mercury may prove, I hope it will in future be employed with more circumſpection,

"*Felix quem faciunt aliena pericula cautum*;"

for although it has not been ſuccefsful in my hands, yet, conſidering the manner in which it has been recommended by ſome men eminent in our profeſſion, I would not wiſh, under certain circumſtances, to diſcountenance its uſe; for where the inflammatory diatheſis is abſent, and a general ſtimulant may be uſeful, there, provided evident marks of the diſeaſe are preſent, I would purſue a mercurial courſe: but as the ſubjects of this diſeaſe are commonly children of the rudeſt health, in whom we often find an inflammatory determination to the head, I would, in ſuch caſes, previouſly to the uſe of mercury, recommend a general evacuating plan.

*Taunton*,
May 19, 1788.

